# Michigan State Board of Health

**Published:** 1882-05

**Authors:** 


### ARTICLE X.

### MICHIGAN STATE BOARD OF HEALTH.

*Reported for the* CHICAGO MEDICAL JOURNAL AND EXAMINER:

The regular quarterly meeting of this Board was held at Greenville, Michigan, on April 11, 1882, in connection with the Sanitary Convention held at the same time and place. The following members were present: Rev. D. C. Jacokes, of Pontiac; J. H. Kellogg, M. D., of Battle Creek; Arthur Hazlewood, M. D., of Grand Rapids; John Avery, M. D., of Greenville; Henry B. Baker, M. D., of Lansing, secretary. William Oldright, M. D., chairman, and J. J. Cassiday, M. D., members of the newly appointed Provincial Board of Health of Ontario, were present, and were invited to take seats in the meeting. In the absence of the president of the board, Dr. Jacokes presided.

The secretary presented the subject of inspection of immigrants, and stated that the National Board of Health had granted the request of this board for an inspection service at

Port Huron, and the system would go into effect on May 1, at which time the whole system, by coöperation of several State Boards of Health, would go into effect. He suggested that the health authorities of Toledo and Cleveland be invited to join in this movement. He stated that at the meeting of the Sanitary Council of the Mississippi Valley, at Cairo, Ill., April 19, this subject would be considered, and that it was desirable that this board be represented at that meeting. By vote of the board, Dr. Baker was requested to represent the board at that meeting. Dr. Oldright spoke of the inspection of immigrants at Toronto, and of the importance of notification to other boards of danger to be feared from immigrants. He also said any movement made by this board would meet with hearty coöperation by the Ontario Board. He said the work done by this board for the restriction of scarlet fever and diphtheria was fully as important as that for the restriction of small-pox.

The following motion was carried:

That the secretary be instructed to correspond with the health authorities of the Dominion of Canada and the several provinces thereof, and of provincial and municipal boards of health where they exist, asking their coöperation in the proposed immigrant inspection service.

Dr. Hazlewood read a proposed document giving best household antidotes to be used in case of poisoning, while waiting for a physician, or when one is not to be had. It was accepted and the committee authorized to modify it before publication in the annual report.

Dr. Hazlewood, Committee on Poisons, etc., presented a letter from Dr. Gordon, of Swartz Creek, relative to lead-poisoning by use of a feeding-bottle (which was exhibited to the board), in which the sinker keeping the supply pipe in the milk, was of lead, and so arranged that all the milk had to pass over it before entering the infant's mouth. The secretary was requested to notify the manufacturer of the pernicious character of the bottle, and the report was accepted and ordered published in the annual report.

Circular 35, revised, relating to the duties of health officers, was presented, adopted and twenty thousand copies ordered printed.

Dr. Kellogg, as special committee to prepare a circular on criminal abortion, made a report and read a proposed circular. The report was accepted, the committee continued, and the subject of issuing the circular laid over.

Dr. Kellogg was requested to represent the board at the meeting of the American Medical Association at St. Paul.

The next meeting of the board will be on Tuesday, July 11, 1882.

